# Study on the Erosion Law and Erosion Prediction Model of Pipe Columns During Gas Storage Reservoir Injection and Production Processes

**DOI:** 10.3390/ma18071510

**Published:** 2025-03-27

**Authors:** Zongxiao Ren, Chenyu Zhang, Wenbo Jin, Xuemei Luo, Zhaoyang Fan, Fan Zhang

**Affiliations:** College of Petroleum Engineering, Xi’an Shiyou University, Xi’an 710065, China; 963829195zcy@gmail.com (C.Z.); jinwenbo725@163.com (W.J.); lxm102528@163.com (X.L.); 13571050478@163.com (Z.F.); fanz@xsyu.edu.cn (F.Z.)

**Keywords:** gas storage, pipe column, erosion rate, orthogonal experiment, curve fitting

## Abstract

There are few studies on erosion problems in gas storage environments. High-speed gas-carrying sand in gas storage wells can cause pipeline erosion and subsequent failure. To this end, a numerical model of gas/liquid/solid three-phase erosion under high temperature and high-pressure conditions in gas storage was established. The model combines the laws of conservation of mass, momentum, and energy, as well as the force model of solid particles. Using the established mathematical model of erosion, numerical simulations were performed to study the erosion process of the column under different parameters during gas injection and extraction to find the main factors affecting column erosion. Subsequently, a prediction model was established based on the determined main factors to estimate the maximum erosion rate of the column. The results show that during the extraction process, the maximum erosion rate is exponentially related to the extraction rate, and the erosion intensifies when it is greater than 1 million m^3^/d. It is linearly related to the particle mass flow rate and the well inclination, and it tends to decrease when the particle size is greater than 3 mm. The erosion law during gas injection is similar to that during extraction, but the erosion during gas injection is more severe. The comprehensive influence of various parameters on the maximum erosion rate was studied by orthogonal experiments, and an erosion prediction model was established by nonlinear regression using the least squares method.

## 1. Introduction

Gas storage reservoirs’ strong injection and extraction characteristics result in the continuous expansion of microfractures within the reservoir. This, in turn, initiates the sand phenomenon in the wellbore [1]. Consequently, high-velocity gas will carry particles, causing severe erosion damage to the tubing column, which will, in turn, affect the water supply of the reservoir. Consequently, it is imperative to simulate the erosion of sand-carrying high-velocity gas, to analyze the erosion influence of different influencing factors on the tubular column in the process of injection and extraction, to ascertain the most severe area of tubular column erosion, and to determine the erosion rate of the tubular column under different working conditions. In recent years, a significant number of scholars have conducted extensive research on the issue of pipe column erosion.

Due to the uniqueness of offshore gas fields, the study of their erosion problems requires the coupling of erosion problems with corrosion problems for research. Guo Minling [2] investigated the problems caused by the coupled action of erosion/corrosion in the anti-sand pipe column of offshore high-production gas wells through indoor experiments and established an empirical model applicable to the coupled action of erosion/corrosion in the screen pipe by considering factors such as CO_2_ partial pressure, temperature, flow rate, grain size, and sand content. Meng Wenbo [3] used a designed high-temperature, high-pressure erosion/corrosion coupling test apparatus to study the erosion/corrosion coupling of N80 steel and 13Cr steel under different working conditions and to test the effects of CO_2_ partial pressure, sand mass fraction, sand grain size, fluid flow rate, and other factors. The results show that the steel with higher resistance to CO_2_ corrosion also has higher resistance to erosion/corrosion coupling. Under CO_2_ partial pressure of 0–5 MPa and a 13Cr erosion/corrosion coupling rate, N80 steel was subjected to very severe corrosion. Compared with the sand mass fraction and sand grain size, the impact flow rate has a greater influence on the coupling rate, and the degree of influence of the sand impact angle on the coupling rate is in the order of 45° > 90° > 0°.

For onshore gas fields and reservoirs, corrosion is less considered, and only erosion is studied. Many indoor erosion experiments and numerical simulation studies have been carried out by previous researchers.

Zhang Nan [4] experimentally simulated the effect of erosion on super 13Cr tubing during fracturing construction and found that when the impact angle is large, erosion is mainly manifested as squeezing and forging, while when the impact angle is small, it is dominated by micro-cutting. The erosion rate increases with increasing fracturing fluid flow rate and sand content, and there is a power function relationship between the erosion rate and injection flow rate. Based on the experimental data, the researcher established a calculation model between the jet flow rate and the erosion rate. Yun Wang [5] investigated the erosion of 13Cr tubes in three-phase gas/liquid/solid flow using indoor experiments. With the increase in solid phase particle concentration, particle size, flow rate, and water content, the erosion rate of 13Cr oil pipe increased; the solid phase particle concentration had the most significant effect on oil pipe erosion. Zhang Zhi [6], based on the inadequacy of the existing pipe column structural integrity standards, conducted a study on the integrity of a pipe column under the dual role of pipe column erosion conditions and load risk. The results show that when the yield is certain, the appropriate selection of the lower part of the pipeline, with a large inner diameter and thin wall thickness, can reduce the critical erosion flow rate and improve the ability of the pipeline to resist erosion. Tan Hao [7] studied the erosion of CT80 oil pipe and N80 steel in liquid/solid two-phase flow through indoor experiments and investigated the impact angle, flow velocity, and sand content as three influencing factors on the erosion rate. The results show that CT80 steel in the impact angle of 30° when the erosion rate is the largest, N80 steel 15° when the largest; N80 steel flow rate from 1~5 m/s, the test rate increase of about 13.2 times for the fastest growing stage, CT80 steel in this flow rate stage increase than the N80 steel small; N80 steel in the sand content from 0. 5 percent to 1 percent of the fastest growth in erosion rate, CT80 steel sand content from 0.5 percent to 1 percent of the fastest growth in erosion rate, and N80 steel sand content from 0.5 percent to 1 percent of the fastest growth in erosion rate. N80 steel sand content increased from 0.5% to 1% of the fastest growth in erosion rate, CT80 steel sand content from 0.5% to 3% of the erosion rate first increased and then reduced the fluctuation trend. Cui Lu et al. [8], through the QT1100 continuous pipeline liquid/solid two-phase flow erosion experiments, studied the sand-carrying liquid scouring angle, scouring speed, sand mass concentration, sand type, and sand particle size on the test steel’s erosion resistance. The results show that avoiding the 45° impact angle and using a sand-carrying medium with a sand concentration of 60 kg/m^3^ can significantly reduce the erosion rate of the pipe. Huang Dequan [9] experimentally investigated the effect of CT110 steel, 2507 stainless steel, and 42CrMo steel on the erosion performance of pipes under different erosion conditions. The results show that at an erosion speed of 23~43 m/s, CT110 steel grade plate erosion increased by 18.82 times; as the erosion time increases, the erosion amount increases significantly but tapers off. At an erosion angle of 15°, the CT110 steel grade plate has an absolute erosion amount of 0.0246 g/min, a rate of 7.455 × 10^−4^ g/(min·m·s^−1^); at 60° and 90°, absolute erosion per unit time and the rate of change is not large. The erosion test shows that 2507 stainless steel erosion resistance is the best, followed by CT110, and 42CrMo is worse.

He Mengqi [10] investigated the influence of gas extraction rate, production pressure, sand discharge rate, and sand particle size on the erosion rate of a tubular column by combining experimental simulation and numerical simulation in a Y-type wellhead of the gas storage reservoir. The study shows that sand discharge rate, gas extraction rate, and production pressure play an important role in the erosion of tubular columns. Based on the results of experiments and numerical modeling, a model for the erosion rate of tubular columns of injection and extraction wells of gas storage reservoirs was established by considering the gas production rate, production pressure, sand production rate, and sand particle size. Yang et al. [11] investigated the effects of the geometry of tubular columns of gas storage reservoirs, the fluid properties, and the type of particles on erosion using indoor experiments and numerical simulations. The study shows that the product of the square of the daily gas production, the negative square of the gas production pressure, and the primary of the sand production rate is directly proportional to the erosion rate of the tubular column, and the critical erosion coefficient (C-value) diagram applicable to the gas storage and production corridor has been established by the design criteria of API RP 14E. Based on numerical simulation, Li Mingxing [12] investigated the influence of gas-solid two-phase flow on the erosion rate of the N80 oil pipeline and discussed the influence of production parameters such as gas extraction volume, gas extraction pressure, and particle properties. The study shows that the maximum erosion rate increases with the increase of gas extraction; the increase of gas extraction pressure reduces the erosion degree of the pipe column, the erosion degree increases linearly with the amount of sand, and the decrease of particle diameter and sphericity will increase the erosion rate. Based on the research focus on the critical erosion rate, the determination of the critical production parameters of the N80 oil pipeline plate. Peng Tao [13] investigated the influence mechanism of particle morphology on the erosion characteristics of gas storage wellbore using the theory of computational fluid dynamics (CFD). The experiment shows that as the surface roughness of the pipe wall increases, the gas phase sand carrying capacity shows an improved trend, while the corresponding abrasion degree is weakened. He Zuqing [14] carried out a simulation of tubular column erosion in injection and production wells based on the API RP 14E standard for the Wen23 gas reservoir. The results show that the actual flow rate and critical erosion rate increase and then decrease with increasing well depth under the gas injection condition, while the flow rate and critical erosion rate decrease and then increase in the gas extraction stage. According to the study, the final gas injection rate of Φ88.9 mm × 6.45 mm injection column should not exceed 95 × 104 m^3^/d. Yanhong Wu [15] used a numerical simulation method to study the injection column and clarified the influence of gas extraction rate, column diameter, gravel particle size, and sand discharge rate on the erosion rate of the column. The results show that the erosion rate is directly proportional to the gas extraction rate and sand discharge rate and inversely proportional to the pipe column diameter; the erosion rate decreases with the increase in gravel diameter, then increases, and then remains basically unchanged. In a simulation of gas storage reservoir injection and extraction column erosion and limit of gas injection and extraction volume design study, Wu Zebing [16] used Fluent software 2022r1 to carry out a numerical simulation of pipeline erosion under different fluid parameters and structural parameters to study the inlet flow rate, particle diameter, mass flow rate, viscosity, and the diameter of the elbow on the erosion of the influence of the law and its curve fitting. The results show that the flow rate, particle diameter, and viscosity are quadratic functions of the maximum erosion rate, and the mass flow rate and elbow diameter are power functions of the maximum erosion rate.

Although a large number of studies have investigated the various factors affecting column erosion, only the offshore erosion/corrosion coupling studies consider high temperature and high-pressure environments, whereas the analyses of the effects of various factors on column erosion under high temperature and high-pressure conditions (40–101 °C, 10–35 MPa) are almost unknown in physical experiments as well as numerical simulation experiments in onshore gas fields and gas storage reservoirs. In addition, research on specific gas storage application scenarios is still in its infancy, and existing models are unable to predict the erosion rate during gas injection and recovery.

The aim of this paper is to fill this research gap by addressing the problem of column erosion under high temperature and high-pressure conditions in gas storage reservoirs. Taking the gas storage well X as the research object, the influence of temperature, pressure, gas withdrawal, and other factors on the erosion behavior of injection and withdrawal tubular columns is investigated by numerical simulation, and the erosion rate prediction model is proposed. The research in this paper not only provides theoretical support for the erosion-resistant design of the tubular column but also has important application value for the safe operation of gas storage reservoirs.

## 2. Mathematical Models

### 2.1. Model Assumption

(1) The content of solid particles is less than 10%.
(2) The incident particles are spherical, independent, and uniform in shape, and collisions between particles and possible binding and destruction of particles during collisions are not considered.
(3) Changes in physical properties due to phase transitions are not considered.
(4) Particles do not slide when in contact with the wall, thus simplifying the calculation of friction and impact load.

### 2.2. Continuous Phase Models

In the late stage of gas storage wells, the process of extraction of the formation water occurs concomitantly with that of the gas. At this time, the fluid system contains gas, liquid, and particle phases concurrently. Therefore, to describe the flow field and temperature field in the column, the Mixture model is used. The model disregards local variability, and the continuity Equation (1), momentum Equation (2), and energy Equation (3) are as follows [17,18]:

(1) $$\frac{\partial \rho_{m}}{\partial t}+\nabla \cdot \left(\rho_{m}{\vec{v}}_{m}\right)=0$$

(2) $$\frac{\partial }{\partial t}\left(\rho_{m}{\vec{v}}_{m}\right)+\nabla \cdot \left(\rho_{m}{\vec{v}}_{m}{\vec{v}}_{m}\right)=-\nabla p+\nabla \cdot \left[\mu_{m}\left(\nabla {\vec{v}}_{m}+{\nabla {\vec{v}}_{m}}^{T}\right)\right]+\rho_{m}g+\vec{F}-\nabla \cdot \left(\sum_{k=1}^{n}\alpha_{k}\rho_{k}{\vec{v}}_{dr,k}{\vec{v}}_{dr,k}\right)$$

(3) $$\frac{\partial }{\partial t}\sum_{k}\left(\alpha_{k}\rho_{k}E_{k}\right)+\nabla \cdot \sum_{k}\left(\alpha_{k}{\vec{v}}_{k}\left(\rho_{k}E_{k}+p\right)\right)=\nabla \cdot \left(k_{eff}\nabla T-\sum_{k}\sum_{j}h_{j,k}{\vec{J}}_{j,k}+\left(\tau_{eff}\cdot \vec{v}\right)\right)+S_{h}$$

where $\rho_{m}$ is the density of the mixture, kg/m^3^; t is the time, s; ${\vec{v}}_{m}$ is the velocity of the mixture, m/s; $\alpha_{k}$ is the volume fraction of phase k; $\mu_{m}$ is the viscosity of the mixture, pa·s; ${\vec{v}}_{dr,k}$ is the drift velocity of sub-phase k, m/s; g is the acceleration of gravity, m/s^2^; $h_{j,k}$ is the enthalpy of substance j in phase k, J; ${\vec{J}}_{j,k}$ is the diffusive flux of substance j in phase k; $k_{eff}$ is the effective thermal conductivity, W/(m·k).

### 2.3. Turbulence Models

The geometric model of the gas storage pipe column is relatively simple; consequently, the reliable k-ε turbulence model is utilized for calculation in this paper to enhance the accuracy of the calculation results. This model has been improved based on the standard k-ε model. In comparison with the standard k-ε model, the reliable k-ε model [19,20,21] has been shown to provide enhanced calculation accuracy, particularly in the boundary layer and secondary flow region, which are more prevalent in the study of gas/solid two-phase flow erosion problems. The model achieves an optimal balance between computational efficiency and accuracy, making it highly suitable for this study. Consequently, the reliable k-ε turbulence model is selected to simulate and analyze the gas storage reservoir pipe column in this paper, and the transport equations of turbulent kinetic energy k and dissipation rate ε are shown as follows:

(4) $$\frac{\partial }{\partial t}\left(\rho k\right)+\frac{\partial }{\partial x_{i}}\left(\rho {\vec{u}}_{j}k\right)=\frac{\partial }{\partial x_{i}}\left[\left(\mu +\frac{\mu_{t}}{p\gamma_{k}}\right)\frac{\partial k}{\partial x_{i}}\right]+G_{k}+G_{b}-\rho \varepsilon -Y_{M}$$

(5) $$\frac{\partial }{\partial t}\left(\rho \varepsilon \right)+\frac{\partial }{\partial x_{j}}\left(\rho {\vec{u}}_{j}\varepsilon \right)=\frac{\partial }{\partial x_{j}}\left[\left(\mu +\frac{\mu_{t}}{p\gamma_{\varepsilon }}\right)\frac{\partial \varepsilon }{\partial x_{j}}\right]+\rho C_{1}{\bar{S}}_{\varepsilon }-C_{2}\rho \frac{\varepsilon^{2}}{k+\sqrt{v\varepsilon }}+C_{\varepsilon 1}\frac{\varepsilon }{k}C_{\varepsilon 3}G_{b}$$

where k is the turbulent kinetic energy, J; ε is the dissipation rate, J/S; ${\vec{u}}_{j}$ is the mean velocity, m/s; $x_{i}$ is the spatial coordinate, mm; $\mu_{t}$ is the turbulent viscosity coefficient; $G_{K}$ is the turbulent kinetic energy generation term; $G_{b}$ is the turbulent kinetic energy generation term caused by buoyancy; $Y_{M}$ is the fluctuating energy; $C_{\varepsilon 1}$ and $C_{\varepsilon 2}$ is the empirical constant.

### 2.4. Discrete Phase Models

In this paper, the focus is on the study of gas/liquid/solid three-phase flow, wherein methane and liquid are regarded as continuous phases, while solid particles are considered discrete entities enveloped in the fluid. The Eulerian method is employed to calculate the continuous phase, while the particle trajectory tracking for the discrete particles is simulated using the Lagrange method. The analysis is underpinned by Newton’s second law, and the resulting force equation for the particles is obtained [22]:

(6) $$\frac{dU_{p}}{dt}=F_{D}\left(U_{f}-U_{p}\right)+\frac{g\left(\rho_{p}-\rho_{f}\right)}{\rho_{p}}$$

(7) FD=18μρpDp2CDRep24

(8) Rep=ρfUf−UpDp/μ

where $F_{D}\left(U_{f}-U_{p}\right)$ is the drag force per unit mass of the particle, N; $U_{f}$ is the gas phase velocity, m/s; $U_{p}$ is the particle velocity, m/s; $D_{p}$ is the particle diameter, mm; Rep is the Reynolds number of the particle; $C_{D}$ is the drag force coefficient, which is related to the change of Reynolds number.

### 2.5. Erosion Models

The erosion model adopts the plastic cutting theory proposed by FINNIE [23] and combines factors such as particle mass flow rate, particle size, fluid properties, etc., and the corresponding model is proposed [24] with the following expression:

(9) $$ER=\sum_{n=1}^{N}\frac{m_{p}C\left(d_{p}\right)f\left(\alpha \right)U_{p}^{b\left(v\right)}}{A_{face}}$$

where ER is the erosion rate, kg/(m^2^·s); $m_{P}$ is the particle mass flow, kg/s; $C\left(d_{p}\right)$ is the particle size function, generally take $1.8\times 10^{-9}$; $f\left(\alpha \right)$ is the impact angle function; $b\left(v\right)$ is the speed function, generally take 2.6; $A_{face}$ is the sand particles impact wall area, m^2^.

### 2.6. Model Validation

**Figure 1:**
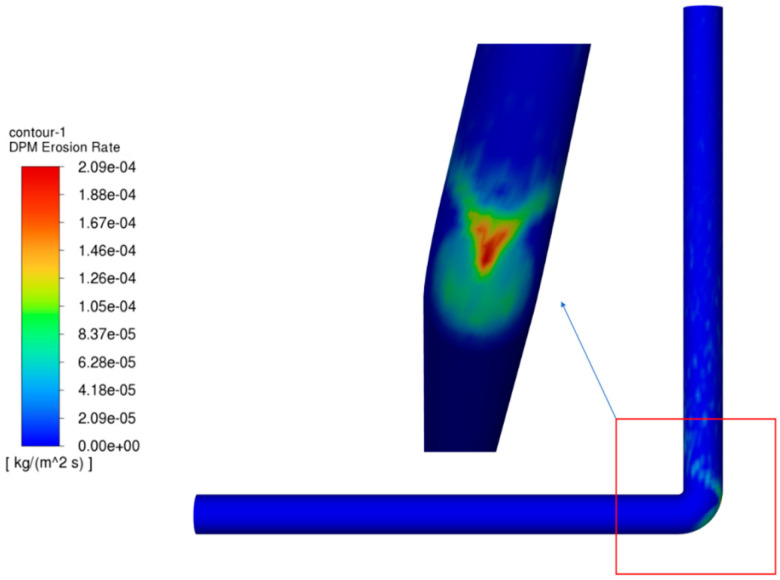
Cloud diagram of validation results.

**Figure 2:**
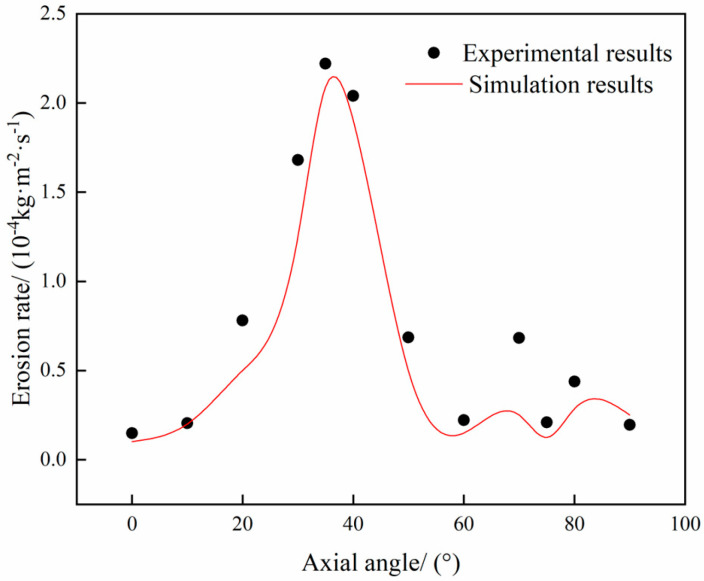
Model Validation Comparison Chart.

To verify the reliability of the numerical model, numerical calculations were carried out using the experimental conditions documented in the literature (see Table 1 for details) [25]. These were then compared with the experimental results. The computational cloud diagrams are shown in Figure 1, and the erosion rate of the outer side of the elbow at different angles is compared with the experimental data, as shown in Figure 2.

The results of the calculation, based on the experimental conditions, indicate that the maximum erosion site is located on the exterior aspect of the elbow, with the erosion concentration area distributed in an elliptical shape (see Figure 2). The erosion rate of the exterior aspect of the elbow at different angles is compared with the experimental data in the figure, and the simulated trend is consistent with the experimental trend. The maximum erosion rate obtained from the simulation is 2.09 × 10^−4^ kg/(m^2^·s), and after conversion, the erosion rate is 0.097 mm/h. Compared with the experimental result of 0.1036 mm/h, the relative error is 6.37%, which meets the requirements of practical engineering applications [26]. Consequently, it can be concluded that the model established in this study can be effectively utilized to predict the erosion rate of the pipe column.

## 3. Geometrical Modeling and Meshing

### 3.1. Geometrical Modeling of Gas Storage Columns

**Figure 3:**
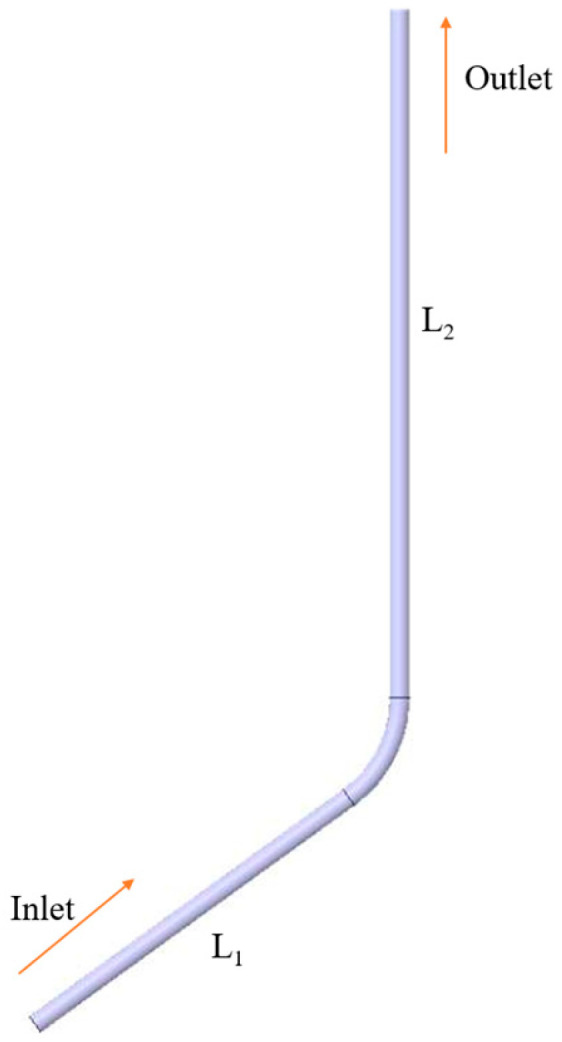
Structure of the pipe column.

The paper uses the example of the Storage X well, which is subject to more severe erosion in the field. The length of the inlet section L_1_ and the length of the outlet section L_2_ are 30 and 40 times the pipe diameter, respectively, and the maximum inclination angle of the well is 27.1°; the structural dimensions are shown in Figure 3, and the fluid parameters in the well are shown in Table 2.

### 3.2. Grid Division and Irrelevance Verification

**Figure 4:**
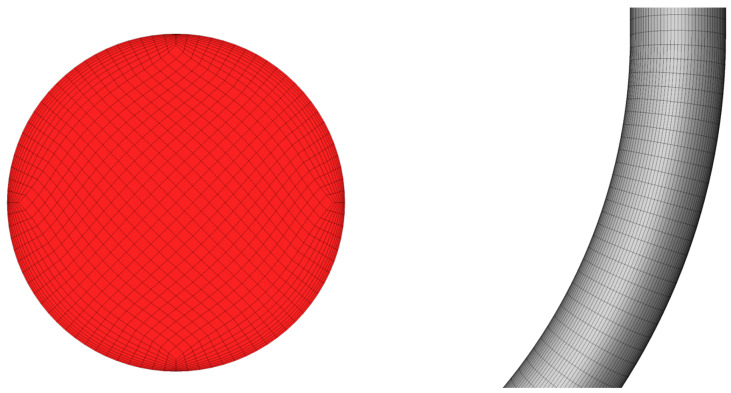
Grid division result diagram.

In this paper, mesh is used for meshing to generate a structured mesh, as shown in Figure 4. Since the fluid flow inside the pipe column belongs to the turbulent state, the influence of the boundary layer must be specially considered during the meshing process to ensure the accuracy of the calculation results. Therefore, in this paper, the number of boundary layers is calculated by setting the y+ value to 30. After calculation, the number of boundary layers obtained is 10, and this setting can effectively ensure the accuracy of the calculation.

Mesh independence validation is a key step in numerical simulation, aiming to ensure that the simulation results are not significantly affected by the mesh delineation, thus verifying the accuracy and reliability of the numerical solutions. By gradually refining the mesh and comparing the calculation results under different mesh conditions, it can be ensured that the numerical solution tends to be stable after the mesh has been refined to a certain extent. In this paper, Fluent is utilized to execute the requisite calculation, with the no-slip condition being adopted for the wall and the standard wall function being employed in the near-wall region. Within the solution process, the SIMPLE algorithm is employed, and the convergence criterion is set as follows: the scale residuals of the energy equation are 10^−6^, and the scale residuals of the other equations are 10^−4^. The wall boundary conditions are set as reflection conditions. The complete setup is shown in Table 3. The specific parameters are shown in Table 4.

**Figure 5:**
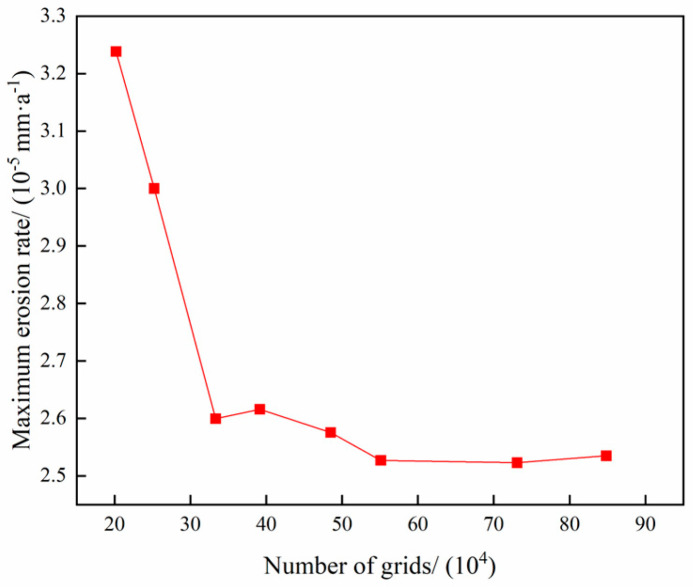
Grid irrelevance analysis.

To verify the mesh independence of the pipe columns, a total of 8 mesh sets with 202,000, 252,096, 333,300, 391,476, 391,476, 484,800, 551,056, 730,836, and 848,400 meshes were divided for the calculation. The results are shown in Figure 5.

From Figure 5, it can be seen that when the number of grids is greater than 551,056, there is no significant change in the maximum erosion rate of the pipe column. Therefore, to save computational cost, the grid number 551,056 is selected for the calculation.

## 4. Single-Factor Analysis and Predictive Model Regression of the Gas Storage Reservoir Column Injection Process

The daily gas production from well X is approximately 5 × 10^4^–60 × 10^4^ m^3^/d, and the grain size range of the sand particles in the field measurement data is 0.01–6 mm. Based on the mathematical model and the geometric model, a sensitivity analysis was carried out on the main factors affecting the erosion of the tubular column, such as gas extraction, particle mass flow rate, particle size, and well inclination angle (the statistics of the gas extraction parameters are shown in Table 5 and the statistics of the gas injection parameters are shown in Table 6, and the bold type are the basic data). By analyzing the relationship between the various factors and the maximum erosion rate of the tubular column, it aims to provide a scientific basis and decision support for subsequent protection measures.

### 4.1. Analysis of the Influence of Gas Injection and Recovery on the Erosion Pattern of Pipe Columns

**Figure 6:**
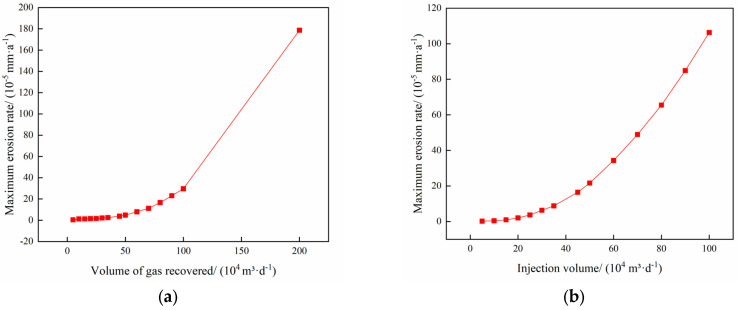
The change rule of maximum erosion rate with gas injection and extraction volume. (**a**) Gas extraction process; (**b**) Gas injection process.

**Figure 7:**
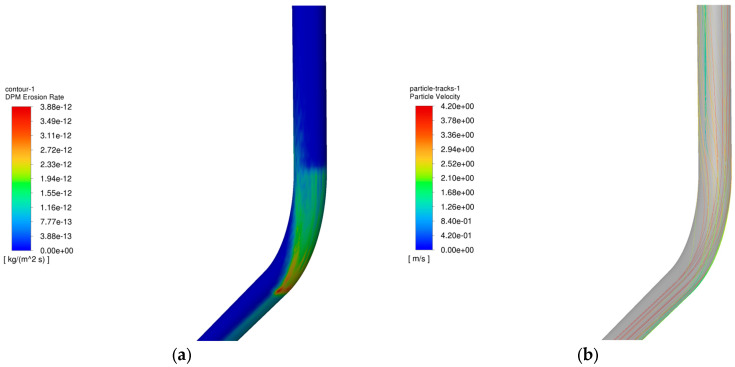

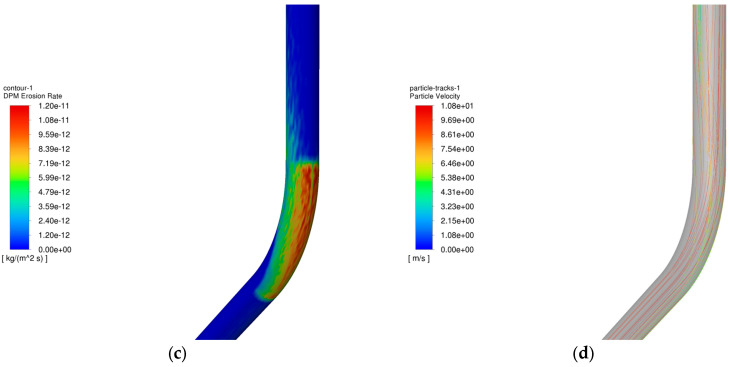
Erosion cloud and particle velocity maps of pipe columns at different gas extraction rates. (**a**) Erosion cloud at 200,000 m^3^/d; (**b**) Particle velocity map at 200,000 m^3^/d; (**c**) Erosion cloud at 500,000 m^3^/d; (**d**) Particle velocity map at 500,000 m^3^/d.

**Figure 8:**
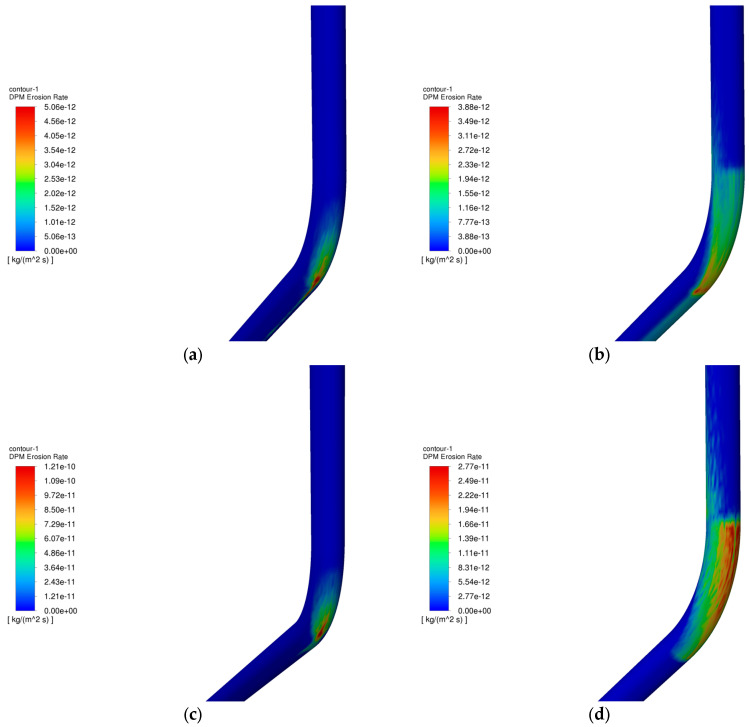
Comparison of column erosion cloud maps during injection and extraction at the same gas volume. (**a**) Erosion cloud at 200,000 m^3^/d gas injection; (**b**) Erosion cloud at 200,000 m^3^/d of gas extraction; (**c**) Erosion cloud at 700,000 m^3^/d gas injection; (**d**) Erosion cloud at 700,000 m^3^/d of gas extraction.

The operating parameters of gas injection and extraction are outlined in Table 3 and Table 4, respectively. Figure 6 demonstrates the relationship between the maximum erosion rate of the pipe column and the volume of injection and extraction gases. Figure 7 presents the erosion cloud and particle velocity diagrams. Figure 8 compares the erosion cloud diagrams of the injection and extraction processes.

Figure 6 shows the variation in the maximum erosion rate with gas injection and extraction volumes. The results show that as the gas volume increases, the particle velocity increases accordingly (see Figure 7b,d), and for the same erosion area, the greater the gas volume, the more severe the erosion of the column (see Figure 7a,c). In addition, there is an exponential relationship between the maximum erosion rate and gas recovery. As shown in Figure 8, the erosion caused by the gas injection process is more significant for the same gas injection and extraction volume, and this result is consistent with existing studies [27]. It can be seen that the maximum erosion rate increases significantly when the gravity is consistent with the flow direction, and the area subject to erosion is more concentrated in the gas injection process compared to the gas extraction process. The main reason for this phenomenon is that the kinetic energy of the particles increases as the gas injection volume increases, resulting in more severe erosion when the particles hit the pipe wall. In addition, because the direction of gas injection is the same as the direction of gravity, the erosion area is more concentrated, further exacerbating the degree of erosion.

### 4.2. Analysis of the Influence of Particle Mass Flow Rate on Column Erosion in the Injection and Extraction Process

**Figure 9:**
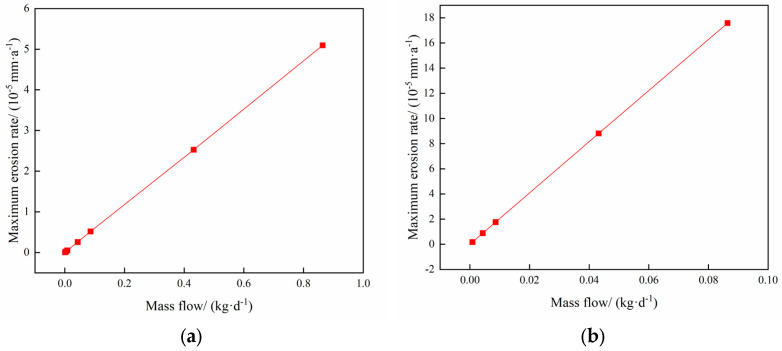
Changing the law of maximum erosion rate with particle mass flow rate in injection mining. (**a**) Gas extraction process; (**b**) Gas injection process.

The operating parameters of injection and withdrawal of gas storage are shown in Table 3 and Table 4, and the rule of variation of the maximum erosion rate of the pipe column with the mass flow rate of particles in the process of injection and withdrawal is shown in Figure 9.

Figure 9 shows the variation of the maximum erosion rate with the mass flow rate. The results show that the greater the particle mass flow rate, the greater the erosion of the column, and the maximum erosion rate is linearly related to the particle mass flow rate. The reason for this is that as the particle mass flow rate increases, the proportion of particles in the fluid increases, and the number of particles receiving kinetic energy increases, making column erosion severe. In addition, during gas injection, an increase in the particle mass flow rate leads to more severe erosion (see Section 4.1 for reasons).

### 4.3. Analysis of the Influence of Particle Size on Column Erosion in the Process of Injection and Extraction

**Figure 10:**
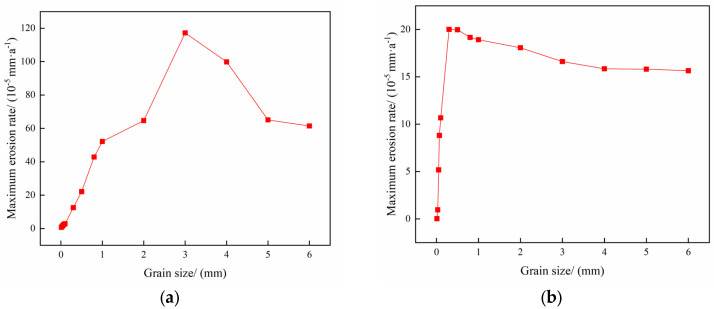
Changes of maximum erosion rate with particle size in injection mining process. (**a**) Gas extraction process; (**b**) Gas injection process.

**Figure 11:**
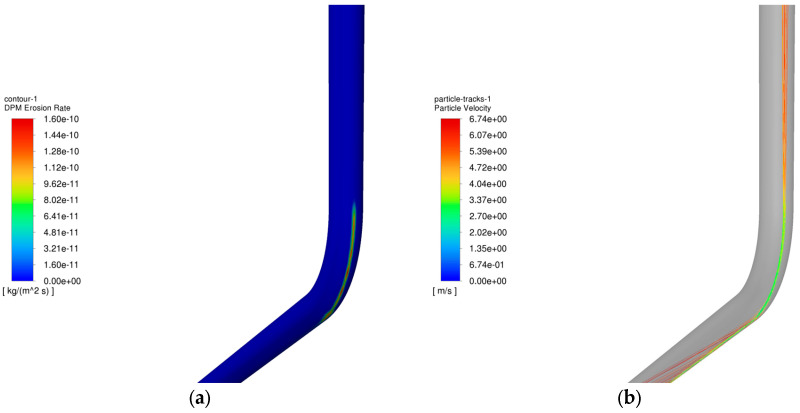

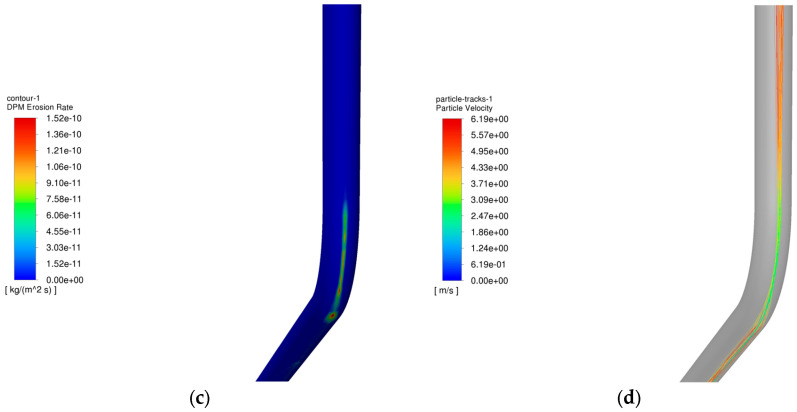
Erosion cloud and particle velocity maps of pipe columns at different grain sizes. (**a**) Erosion cloud at 2 mm; (**b**) Particle velocity diagram at 2 mm; (**c**) Erosion cloud at 6 mm; (**d**) Particle velocity diagram at 6 mm.

**Figure 12:**
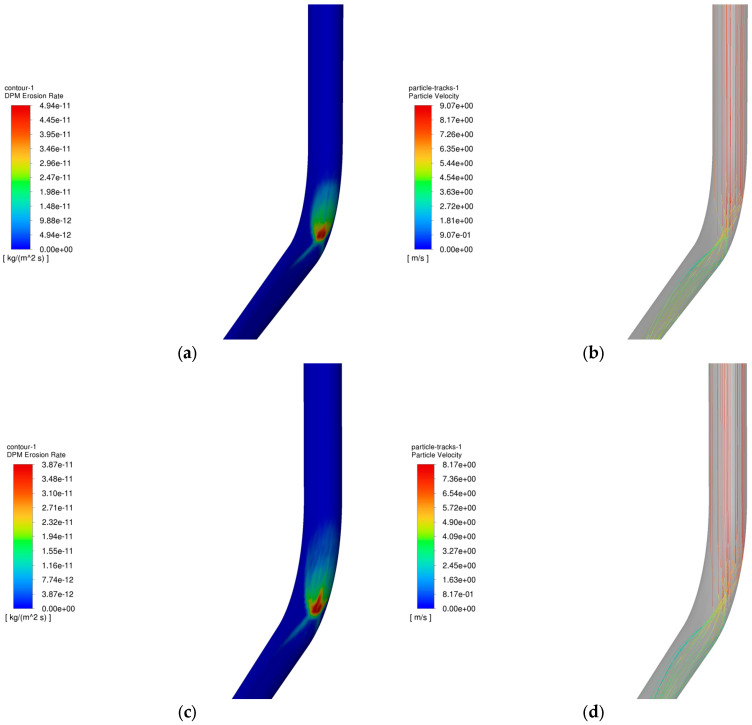
Erosion cloud and particle velocity maps of pipe columns with different particle sizes. (**a**) Erosion cloud at 0.5 mm; (**b**) Particle velocity diagram at 0.5 mm; (**c**) Erosion cloud at 6 mm; (**d**) Particle velocity diagram at 6 mm.

The operating parameters of gas storage injection and extraction are shown in Table 3 and Table 4, and the variation rule of the maximum erosion rate of the pipe column with the particle size in the process of injection and extraction is shown in Figure 10, and the erosion cloud diagram and the particle velocity diagram are shown in Figure 11, and the comparison of the erosion cloud diagrams in the process of injection and extraction is shown in Figure 12.

Figure 10 shows the variation of the maximum erosion rate with particle size during injection and removal. The results show that as the particle size increases, the erosion of the pipe column tends to increase and then decrease. The reason is that as the particle size increases, the fluid velocity is always higher than the particle velocity, the fluid has a carrying effect on the particles, and the kinetic energy of the particles increases, which intensifies the erosion. However, in the process of gas extraction, when the particle size is more than 3 mm, the particle velocity begins to decline (see Figure 11), thus reducing the erosion. Figure 12 shows that when the particle size is 0.5 mm, under the effect of gravity and gas injection, the particles hit the wall of the pipe in a more concentrated area, forming an “inverted V-type” erosion area; as the particle size continues to increase, the particle velocity is gradually affected by its intrinsic properties and reduced, and the degree of erosion is also reduced. The maximum erosion rate in the gas injection process is lower than that in the gas extraction process because the force on the particles is greater in the gas injection process and the particles are more easily broken, leading to a reduction in the impact on the wall, resulting in a lower maximum erosion rate in the gas injection process than the maximum erosion rate in the gas extraction process.

### 4.4. Analysis of the Influence of Well Inclination Angle on the Erosion Pattern of the Tubular Column in the Process of Injection and Extraction

**Figure 13:**
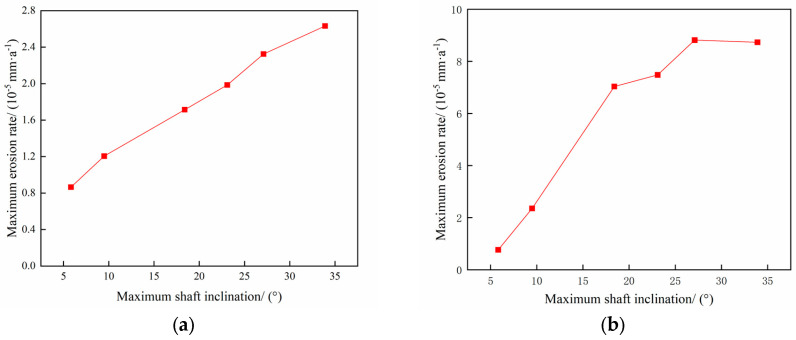
Changing the law of maximum erosion rate with well inclination angle in the injection process. (**a**) Gas extraction process; (**b**) Gas injection process.

**Figure 14:**
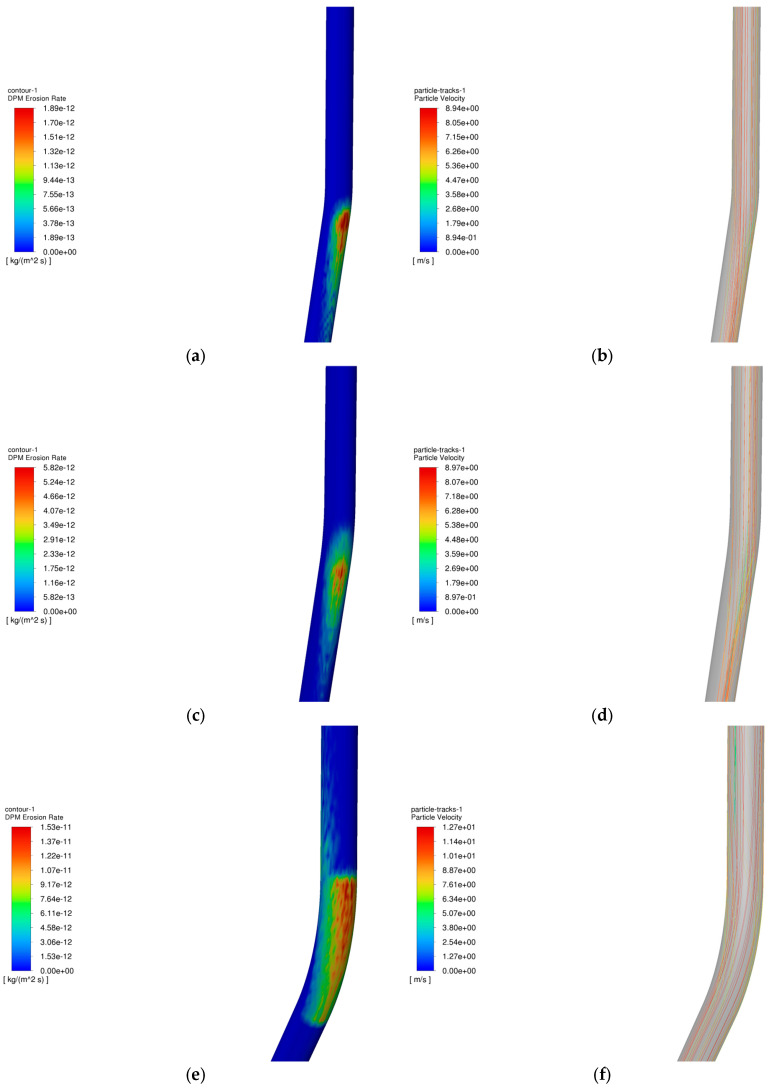
Erosion cloud and particle velocity maps of tubular columns at different well inclination angles. (**a**) Erosion cloud at 5.83°; (**b**) Particle velocity diagram at 5.83°; (**c**) Erosion cloud at 9.5°; (**d**) Particle velocity diagram at 9.5°; (**e**) Erosion cloud at 18.4°; (**f**) Particle velocity diagram at 18.4°.

**Figure 15:**
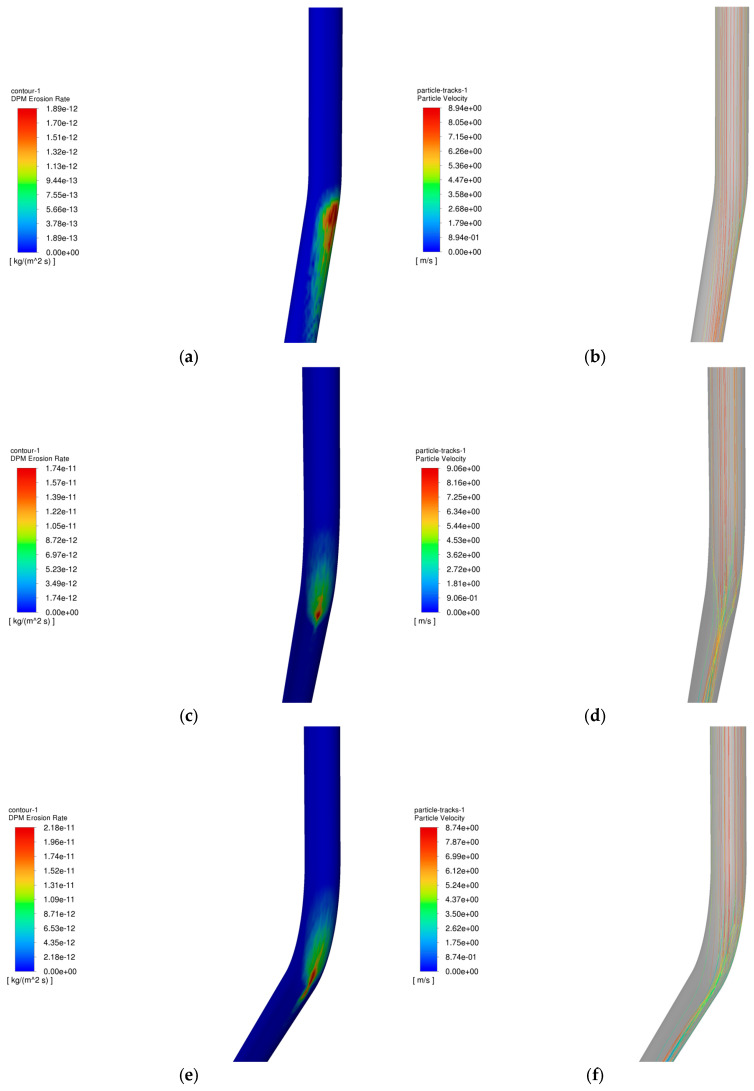
Erosion cloud and particle velocity maps of tubular columns at different well inclination angles. (**a**) Erosion cloud at 5.83°; (**b**) Particle velocity diagram at 5.83°; (**c**) Erosion cloud at 18.4°; (**d**) Particle velocity diagram at 18.4°; (**e**) Erosion cloud at 27.1°; (**f**) Particle velocity diagram at 27.1°.

The operating parameters of gas storage injection and extraction are shown in Table 3 and Table 4, and the change rule of maximum erosion rate of pipe column with particle size in the process of injection and extraction is shown in Figure 13, and the erosion cloud diagram and particle velocity diagram are shown in Figure 14, and the comparison of erosion cloud diagram in the process of injection and extraction is shown in Figure 15.

Figure 13 shows the pattern of maximum erosion rate with well inclination angle during injection and withdrawal. The results show that as the well inclination angle increases, the erosion rate of the tubular column increases slightly. As shown in Figure 14b, when the well inclination angle is small, most of the particles can pass smoothly without colliding with the pipe wall. As shown in Figure 14d,f, more particles collide with the wall as the well inclination angle increases, and the erosion area of the tubular column is gradually enlarged as the well inclination angle increases, leading to an increase in the erosion degree of the tubular column. In the process of gas injection, as the well inclination angle increases, the number of particles colliding with the wall under the effect of gravity and gas injection will increase, and the erosion area will become more concentrated (as shown in Figure 15a,c,e), leading to a further increase in the degree of erosion of the tubular column.

### 4.5. Analysis of the Influence of Temperature and Pressure on Column Erosion in the Process of Gas Extraction

**Figure 16:**
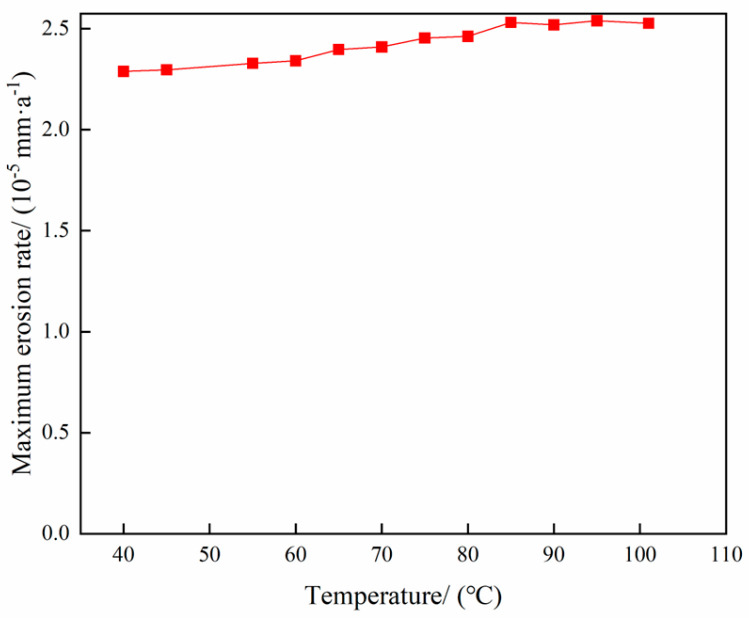
Variation rule of maximum erosion rate with temperature.

**Figure 17:**
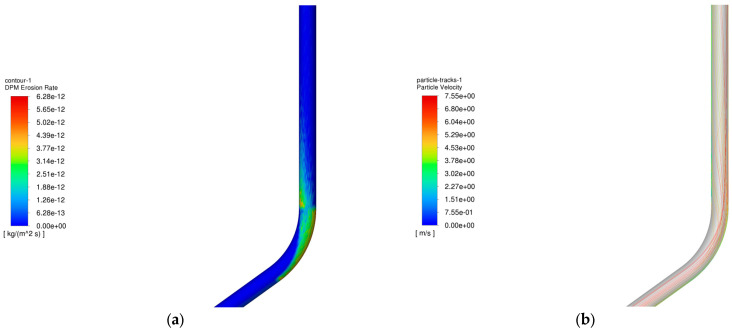

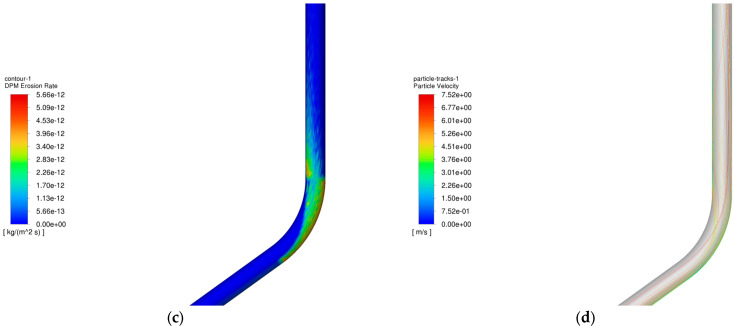
Erosion cloud and particle velocity maps of pipe columns at different temperatures. (**a**) Erosion cloud at 95 °C; (**b**) Particle velocity diagram at 95 °C; (**c**) Erosion cloud at 40 °C; (**d**) Particle velocity diagram at 40 °C.

**Figure 18:**
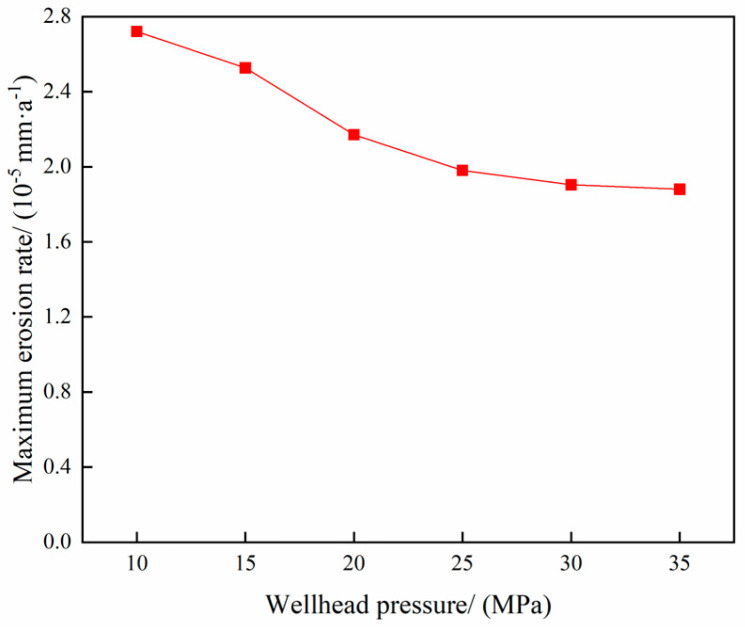
Variation rule of maximum erosion rate with wellhead pressure.

**Figure 19:**
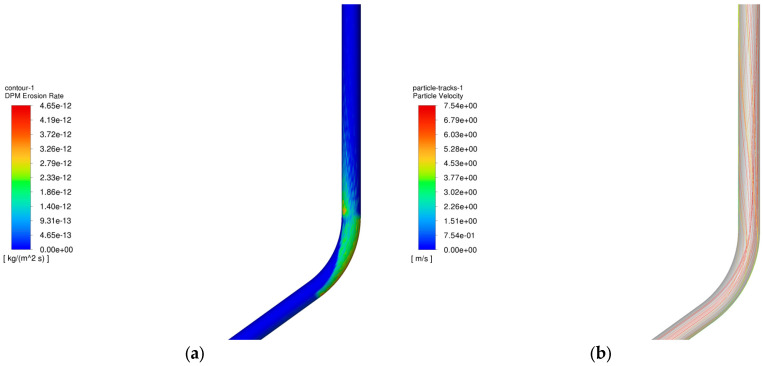

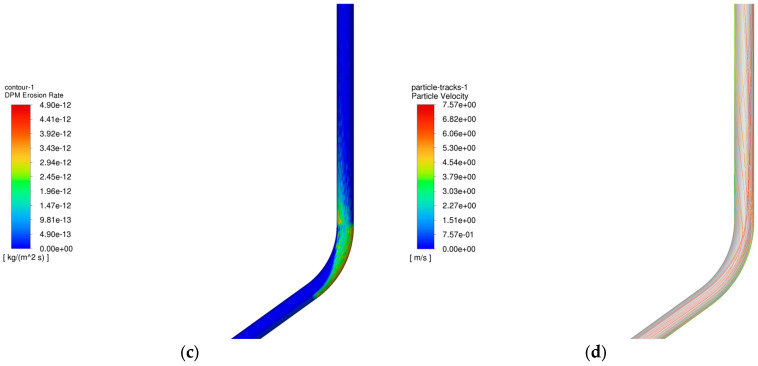
Erosion cloud and particle velocity maps of tubular columns at different wellhead pressures. (**a**) Erosion cloud at 35 MPa; (**b**) Particle velocity diagram at 35 MPa; (**c**) Erosion cloud at 25 MPa; (**d**) Particle velocity diagram at 25 MPa.

The parameters of the gas storage reservoir gas extraction operation are delineated in Table 3. The variation in the maximum erosion rate of the tubing column with temperature is illustrated in Figure 16, while the erosion cloud diagram is presented in Figure 17. The relationship between the maximum erosion rate of the tubing column and wellhead pressure is demonstrated in Figure 18, accompanied by the erosion cloud diagram in Figure 19.

As demonstrated in Figure 16 and Figure 18, the relationship between the maximum erosion rate and temperature and wellhead pressure, respectively, is not a simple one. The findings indicate that the impact of temperature and pressure on erosion is relatively negligible. A decrease in temperature results in reduced erosion to the tubular column, while a decrease in wellhead pressure leads to an increase in erosion. Conversely, an increase in temperature causes a decrease in gas density, increasing the volumetric flow rate and, consequently, an increase in the gas flow rate, thereby exacerbating the erosion damage. Conversely, lower temperatures have been shown to mitigate erosion (see Figure 17b,d). The reduction in particle velocity is smaller when the temperature is lowered, thus not causing significant damage. Concurrently, the decline in wellhead pressure engenders an augmentation in the pressure differential between the two extremities of the tubular column, thereby enhancing the flow rate of the fluid within the tubular column (refer to Figure 19b,d). This, in turn, leads to an escalation in the kinetic energy of the particles, which in turn exacerbates the erosion of the tubular column. However, there is no substantial alteration in the erosion area.

### 4.6. Analysis of the Effect of Water Volume Fraction on Column Erosion During Gas Extraction

**Figure 20:**
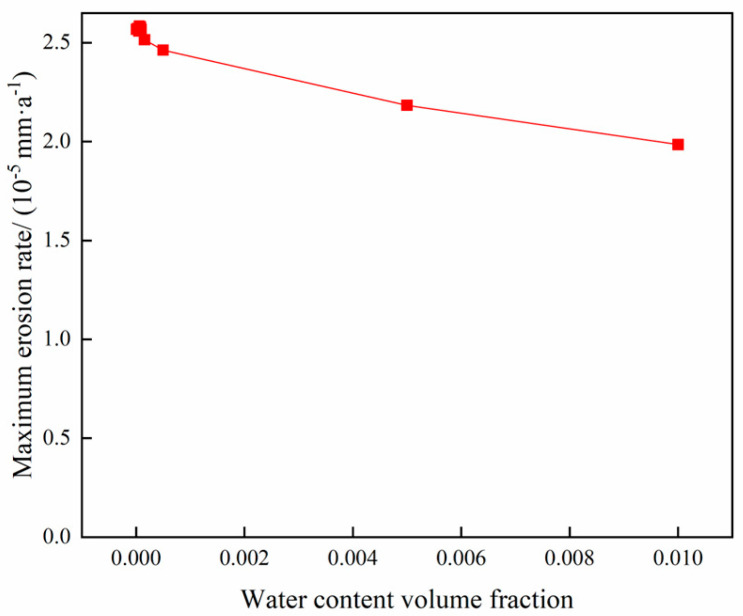
Variation pattern of maximum erosion rate with water content volume fraction.

The operating parameters of gas storage reservoirs are outlined in Table 3, while the variation in the maximum erosion rate of the pipe column with the water content volume fraction is demonstrated in Figure 20. The water content volume fraction ranges from 0.00001 to 0.01, respectively.

As demonstrated in Figure 20, the maximum erosion rate is observed to vary with the water content volume fraction. The findings demonstrate that the maximum erosion rate exhibits a slight decrease with an increase in the water content volume fraction. This phenomenon can be attributed to the observation that an increase in the water content volume fraction of the tube leads to a reduction in gas velocity. The reduction in gas velocity is due to the fact that the liquid reduces the interaction between the sand particles and the gas phase, thereby slowing the movement of sand particles in the gas phase and reducing their collisions with the tube wall. This, in turn, results in a decrease in the erosion rate.

### 4.7. Erosion Rate Prediction Modeling

In the context of engineering, instances of erosion occurring during gas injection are seldom observed. Consequently, the present study has focused exclusively on conducting an erosion model regression analysis for the gas extraction process to provide a reference point for other gas storage reservoirs. The findings of the aforementioned single-factor analysis indicate that the predominant influencing factors of column erosion encompass gas extraction, particle diameter, particle mass flow rate, and well slope angle. To this end, an orthogonal experimental design was developed, covering four factors (flow rate, particle mass flow rate, particle size, and well inclination) and five levels (see Table 7). This design was employed to ensure the experimental data were adequately represented and the regression model was calibrated.

In accordance with the orthogonal experimental data, a predictive model for the erosion rate of the pipe column was obtained by means of least squares fitting.

(10) $$ER=\frac{2.189\times 10^{-8}u^{1.19}m_{p}^{0.75}d_{p}^{0.32}\theta^{1.97}}{1-0.02u-0.0043m_{p}-0.044d_{p}+0.09\theta }$$

where u is the gas extraction volume, 10^4^ m^3^/d; $m_{p}$ is the particle mass flow rate, kg/d; $d_{p}$ is the particle size, mm; θ is the well slope angle, °.

The R^2^ value of the regression equation is 0.962, which indicates that the equation is a good fit. As demonstrated in Table 8, a comparison was made between the results of the indoor erosion simulation experiments and those of the predictive model.

The mean relative error was utilized to evaluate the precision of the experimental data about the calculated outcomes of the fitted model, which was expressed as

(11) $$MRE\;=\frac{1}{n}\sum \frac{\left|R_{predicted}-R_{\exp erimental}\right|}{R_{\exp erimental}}$$

where $R_{predicted}$ denotes the experimental results in the dataset, $R_{\exp erimental}$ signifies the predicted results in the dataset, and n represents the sample size.

From the above calculations, the average relative error between the experimental data and the fitted model is 7.35%, which is within the acceptable range of 10% error for the erosion problem in industry [28].

## 5. Conclusions

In this paper, the erosion problem of gas storage well X in the process of injection and production is studied, a three-phase erosion mathematical model of the reservoir is established, and a sensitivity analysis of the factors in the process of injection and production is carried out to obtain the key factors affecting the erosion of the tubing column, and an erosion rate prediction model for these factors is established.

1. In the process of gas extraction, the erosion of the tubing column is influenced by several factors. The study shows that the larger the gas extraction volume, the more serious the erosion, and the two have an exponential relationship; the larger the mass flow rate of particles, the more significant the erosion, which has a linear relationship; as the particle size increases, the erosion first increases and then decreases; the larger the angle of well inclination, the higher the degree of erosion; the volume fraction of water content slightly reduces the maximum rate of erosion; and the temperature and wellhead pressure have little effect on erosion.
2. In the gas injection process, the erosion law of the pipe column is similar to that of the gas extraction process, but the degree of erosion in the gas injection process is higher than that of the gas extraction process. This is mainly because in the gas injection process, the particles under the action of gravity and airflow to obtain the kinetic energy is greater than in the gas extraction process, and at the same time, the location of the erosion effect of the particles in the gas injection process is more concentrated, resulting in more severe erosion damage.
3. Under the single-factor analysis, the principal controlling factors of tubular column erosion were ascertained to be gas extraction, particle mass flow rate, particle size, and well slope angle. Consequently, the influential factors were analyzed through orthogonal experiments, and a prediction model for the erosion rate of gas storage reservoir tubing columns was established by the least squares method. The relative error between the predicted and simulated values is minimal, thereby validating the adequacy of the prediction model. The established prediction formula can be used as an empirical formula to predict the erosion rate under different gas extraction, particle size, particle mass flow rate, and well inclination angle, thus avoiding the time-consuming and complicated numerical calculations using Fluent.
4. The shortcoming of this paper is that the erosion prediction model is established by numerical simulation and verified by physical experiments, but it has not been verified by field data. The next step will be to optimize the erosion model in combination with field data to obtain a model suitable for the field.

## Figures and Tables

**Table 1:** Physical parameters and results.

Pipe Diameter/mm	Aspect Ratio	Sand Density/(kg·m^−3^)	Pipe Density/(kg·m^−3^)	Fluid Velocity/(m·s^−1^)	Sand Particle Size/mm	Maximum Erosion Rate /(mm·h^−1^)		Sand Mass Flow Rate/(kg·s^−1^)
						Simulation Results	Experimental Results	
** 41 **	** 1.25 **	** 2650 **	** 7800 **	** 25.24 **	0.1	0.097	0.1036	0.015

**Table 2:** Column size and medium parameters.

Column Parameters	Values
Column inner diameter d_i_/mm	76
Column outer diameter d_o_/mm	88.9
well, inclination angle α/°	27.1
Tube density ρ/kg·m^−3^	7800

**Table 3:** Boundary conditions and solver settings.

Project	Project Name	Content
Boundary condition	Entrance boundary	Velocity
	Export boundary	Pressure
	Wall	Standard wall function
Calculation settings	Solver	Pressure-based solvers
	Multiphase flow model	Mixture model
	Turbulence modeling	Realizable k-ε Model
	Solver algorithms	SIMPLE
Spatial discretization method	Gradient term	The least squares method based on control bodies
	Pressure term	PRESTO
	Momentum term	First order windward
	Volume fraction term	Geometric reconstruction
	Turbulent momentum term	Second order windward
	Turbulent dissipation rate term	Second order windward

**Table 4:** Boundary condition parameter setting.

Medium Parameters	Values
incidence velocity v/(m·s^−1^)	6.6
$\mathrm{mass}\;\mathrm{flow}\;\mathrm{rate}\;m_{p}$/(kg·d^−1^)	0.432
particle diameter d_p_/mm	0.07
particle density ρ/kg·m^−3^	2650

**Table 5:** Statistics of gas extraction parameters.

Parameters	Values
Gas production (10^4^ m^3^/d)	5, 10, 15, 20, 25, 30, **35**, 45, 50, 60, 70, 80, 90, 100, 150, 200
Grain size of the sand (mm)	0.01, 0.03, 0.05, **0.07**, 0.1, 0.3, 0.5, 0.8, 1, 2, 3, 4, 5, 6
Sand density (kg/m^3^)	2650 kg/m^3^
Sand mass flow rate (kg/d)	0.000864, 0.00432, 0.00864, 0.0432, 0.0864, **0.432**, 0.864
Well inclination angle (°)	5.83, 9.5, 18.4, 23.1, **27.1**, 33.9
Wellhead pressure (MPa)	35, 30, 25, 20, **15**, 10
Inlet temperature (℃)	**101**, 95, 90, 85, 80, 75, 70, 65, 60, 55, 50, 45, 40

**Table 6:** Statistics of gas injection parameters.

Parameters	Values
Injected quantity (10^4^ m^3^/d)	5, 10, 15, 20, 25, 30, **35**, 45, 50, 60, 70, 80, 90, 100, 200
Grain size of the sand (mm)	0.01, 0.03, 0.05, **0.07**, 0.1, 0.3, 0.5, 0.8, 1, 2, 3, 4, 5, 6
Sand density (kg/m^3^)	2650 kg/m^3^
Sand mass flow rate (kg/d)	0.000864, 0.00432, 0.00864, **0.0432**, 0.0864
Well inclination angle (°)	5.83, 9.5, 18.4, 23.1, **27.1**, 33.9

**Table 7:** Tube column orthogonal experiment table and results.

Number	Flow Rate (10^4^ m^3^/d)	Mass Flow (kg/d)	Grain Size (mm)	Well Inclination Angle (°)	Maximum Erosion Rate 10^−5^ (mm/a)
1	10	0.000864	0.07	5.83	0.0001
2	10	0.0864	3	9.5	0.1027
3	10	0.864	0.5	18.4	3.3841
4	10	0.00864	5	27.1	0.0473
5	10	0.432	1	33.9	3.5175
6	20	0.864	3	33.9	18.8407
7	20	0.00864	0.5	5.83	0.0105
8	20	0.432	5	9.5	10.2290
9	20	0.000864	1	18.4	0.0118
10	20	0.0864	0.07	27.1	0.4447
11	35	0.432	0.5	27.1	52.1557
12	35	0.000864	5	33.9	0.1027
13	35	0.0864	1	5.83	0.8207
14	35	0.864	0.07	9.5	2.6159
15	35	0.00864	3	18.4	1.2331
16	45	0.0864	5	18.4	25.1884
17	45	0.864	1	27.1	64.6892
18	45	0.00864	0.07	33.9	0.3133
19	45	0.432	3	5.83	27.8972
20	45	0.000864	0.5	9.5	0.0280
21	60	0.00864	1	9.5	0.6509
22	60	0.432	0.07	18.4	6.1859
23	60	0.000864	3	27.1	0.7763
24	60	0.0864	0.5	33.9	10.1481
25	60	0.864	5	5.83	137.0603

**Table 8:** Erosion test results and model predictions.

Case	u (10^4^ m^3^/d)	m_p_ (kg/d)	d_p_ (mm)	Θ (°)	Experiment Data/(mm·a^−1^)	Erosion Model Prediction Results/(mm·a^−1^)
1	25	86.4	0.07	27.1	0.0032	0.0031
2	35	86.4	0.07	27.1	0.0050	0.0051
3	45	86.4	0.07	27.1	0.0080	0.0075
4	60	86.4	0.07	27.1	0.0159	0.0142
5	45	43.2	0.07	27.1	0.0037	0.0041
6	45	172.8	0.07	27.1	0.0147	0.0152
7	45	86.4	0.1	27.1	0.0095	0.0084
8	50	86.4	0.07	27.1	0.0089	0.0097
9	35	259.2	0.07	27.1	0.01453	0.0169
10	60	259.2	0.05	27.1	0.046495	0.0471

## Data Availability

The original contributions presented in this study are included in the article. Further inquiries can be directed to the corresponding author.

## Nomenclature

$\rho_{m}$	the density of the mixture	${\vec{J}}_{j,k}$	the diffusive flux of substance j in phase k
${\vec{v}}_{m}$	the velocity of the mixture	$k_{eff}$	the effective thermal conductivity
$\alpha_{k}$	the volume fraction	k	the turbulent kinetic energy
$\mu_{m}$	the viscosity of the mixture	ε	the dissipation rate
${\vec{v}}_{dr,k}$	the drift velocity of sub-phase k	${\vec{u}}_{j}$	the mean velocity
$h_{j,k}$	the enthalpy of substance j in phase k	$x_{i}$	the spatial coordinate
$G_{K}$	the turbulent kinetic energy generation term	$\mu_{t}$	the turbulent viscosity coefficient
$G_{b}$	the turbulent kinetic energy generation term caused by buoyancy	$Y_{M}$	the fluctuating energy
$C_{\varepsilon 1}$	the empirical constant	$C_{\varepsilon 2}$	the empirical constant
$F_{D}\left(U_{f}-U_{p}\right)$	the drag force per unit mass of the particle	$U_{f}$	the gas phase velocity, m/s
$U_{p}$	the particle velocity	$D_{p}$	the particle diameter
Rep	the Reynolds number of the particle	$C_{D}$	The drag force coefficient
ER	the erosion rate	$m_{p}$	the particle mass flow
$C\left(d_{p}\right)$	the particle size function	$f\left(\alpha \right)$	the impact angle function
$b\left(v\right)$	The speed function	$A_{face}$	The sand particles impact the wall area.
di	Column inner diameter	v	incidence velocity v/(m·s^−1^)
$d_{o}$	Column outer diameter	$m_{p}$	mass flow rate
$a{/}^{\circ }$	well inclination angle	$d_{p}$	particle diameter
σ/kg·m^−3^	Tube Density	σ/kg	particle density
u	the gas extraction volume	$m_{p}$	The particle mass flow rate
$d_{p}$	the particle size	θ	the well slope angle°
$R_{predicted}$	The experimental results in the dataset	$R_{experimental}$	The predicted results in the dataset
n	represents the sample size		
